# The emergence of Fanconi anaemia type S: a phenotypic spectrum of biallelic *BRCA1* mutations

**DOI:** 10.3389/fonc.2023.1278004

**Published:** 2023-12-11

**Authors:** Tirion Hughes, Anna M. Rose

**Affiliations:** ^1^ University of Oxford Medical School, Oxford, United Kingdom; ^2^ Department of Paediatrics, University of Oxford, Oxford, United Kingdom

**Keywords:** BRCA1, Fanconi anaemia, DNA damage response, familial cancer syndromes, breast cancer, ovarian cancer, cancer predisposition

## Abstract

BRCA1 is involved in the Fanconi anaemia (FA) pathway, which coordinates repair of DNA interstrand cross-links. FA is a rare genetic disorder characterised by bone marrow failure, cancer predisposition and congenital abnormalities, caused by biallelic mutations affecting proteins in the FA pathway. Germline monoallelic pathogenic *BRCA1* mutations are known to be associated with hereditary breast/ovarian cancer, however biallelic mutations of *BRCA1* were long predicted to be incompatible with embryonic viability, hence *BRCA1* was not considered to be a canonical FA gene. Despite this, several patients with biallelic pathogenic *BRCA1* mutations and FA-like phenotypes have been identified – defining a new FA type (FA-S) and designating *BRCA1* as an FA gene. This report presents a scoping review of the cases of biallelic *BRCA1* mutations identified to date, discusses the functional effects of the mutations identified, and proposes a phenotypic spectrum of *BRCA1* mutations based upon available clinical and genetic data. We report that this FA-S cohort phenotype includes short stature, microcephaly, facial dysmorphisms, hypo/hyperpigmented lesions, intellectual disability, chromosomal sensitivity to crosslinking agents and predisposition to breast/ovarian cancer and/or childhood cancers, with some patients exhibiting sensitivity to chemotherapy. Unlike most other types of FA, FA-S patients lack bone marrow failure.

## Introduction

Fanconi anaemia (FA) is a rare disorder characterised by progressive bone marrow failure (BMF), congenital dysmorphisms (including short stature, microcephaly, skin pigmentation abnormalities, thumb/radial ray malformations), cancer predisposition and hypersensitivity to DNA crosslinking agents (1, 2). FA presents with significant phenotypic variability, even between members of the same family with the same FA-associated mutation (3). A diagnosis of FA is often considered on the basis of BMF alongside congenital dysmorphisms. Diagnosis is usually confirmed by demonstration of *in vitro* chromosomal sensitivity to crosslinking agents, such as DEB (diepoxybutane) (4) or MMC (mitomycin C) (5). Following this, genetic testing can identify which FA-associated gene is mutated (6).

Mutation in twenty-two genes have been identified in FA: *FANCA*, *FANCB*, *FANCC*, *FANCD1/BRCA2, FANCD2, FANCE, FANCF, FANCG/XRCC9*, *FANCI, FANCJ/BRIP1, FANCL, FANCM, FANCN/PALB2, FANCO/RAD51C, FANCP/SLX4, FANCQ/ERCC4/XPF, FANCR/RAD51, FANCS/BRCA1, FANCT/UBE2T, FANCU/XRCC2, FANCV/REV7/MAD2L2*, and *FANCW/RFWD3* (2). *FANCH* was identified as an FA gene but later found to be analogous to *FANCA* (7). The protein products of these genes interact to repair interstrand crosslinks (ICL) by regulating/directing nucleolytic incision, translesion synthesis, and homologous recombination (8). The FA core complex recognises ICLs at a stalled replication fork and ubiquitinates the FANCD2-FANCI (ID2) complex, which recruits downstream effectors of the FA pathway including FANCS/BRCA1. These mediate repair by nucleolytic incision, ICL unhooking, generation of double-strand breaks (DSB), and RAD51-dependent strand invasion and recombination (2, 8). Biallelic pathogenic mutations in these genes cause FA, hence inheritance follows an autosomal recessive pattern, with two exceptions: heterozygous pathogenic variants of *FANCR/RAD51* cause autosomal dominant FA-R (9, 10) and hemizygous pathogenic variants of *FANCB* cause X-linked FA-B (11). *FANCA*, *FANCC* and *FANCG* mutations account for ~85% of FA cases (2), whereas *FANCV* and *FANCW* mutations have only been identified in one FA patient each (12–14).

Whilst biallelic (homozygous or compound heterozygous) mutations in FA-associated genes can result in FA, germline monoallelic mutations in many of these same genes may confer increased cancer risk. For example, biallelic mutations to *BRCA2* underlie FA-D1, whereas monoallelic mutations are frequently observed in hereditary breast and ovarian cancer (HBOC), highlighting the link between FA and BRCA DNA repair pathways (15–17). Further, biallelic mutations in *PALB2* and *BRIP1* are associated with FA-N and FA-J respectively (18–20), but are associated with moderate HBOC risk in a heterozygous setting (21, 22). Despite identification of interaction between BRCA1 and known FA proteins (23), *BRCA1* was not generally considered to be a canonical FA gene, as viable biallelic mutations affecting *BRCA1* were not observed or expected. Mouse models demonstrated that most combinations of *Brca1* biallelic mutations result in embryonic lethality and that one wild-type allele is required for development (24–27). However, there have now been a number of individual case reports of biallelic pathogenic *BRCA1* mutations, many of whom have been identified as having a new form of Fanconi Anaemia – FA-S. This work conducted a scoping review in order to collate all reported cases of biallelic *BRCA1* mutation and FA-S, to allow assimilation of clinical and genetic data on this rare condition.

## Methods

A scoping review was performed by a standardized method. Two databases were used for the search, Medline and SCOPUS. In addition, a grey literature search was conducted using Google Scholar. On each, a standardised search string was used to search for human case reports of germline biallelic *BRCA1* mutations:

- MEDLINE: (brca1 AND (homozygous OR biallelic OR (compound AND heterozygous))).ti.
- SCOPUS: Title((brca1 AND (homozygous OR biallelic OR (compound AND heterozygous))).
- Google Scholar: allintitle: brca1 homozygous OR biallelic OR “compound heterozygous”.

The following exclusion criteria were used:

1. Not reporting a human case of germline biallelic *BRCA1* mutations due to:
  - No human case reported.
  - Involving a gene other than *BRCA1*.
  - Not germline biallelic mutations including:
    i. Multiple *BRCA1* mutations in *cis* rather than in *trans*.
    ii. Non-germline mutations i.e. one or more somatic mutation sequenced in tumours.
2. Duplicates or other clear reasons for exclusion.

Data from included articles was collected in a standardised data collection table to ensure uniformity of data collection.

## Results

**Figure 1:**
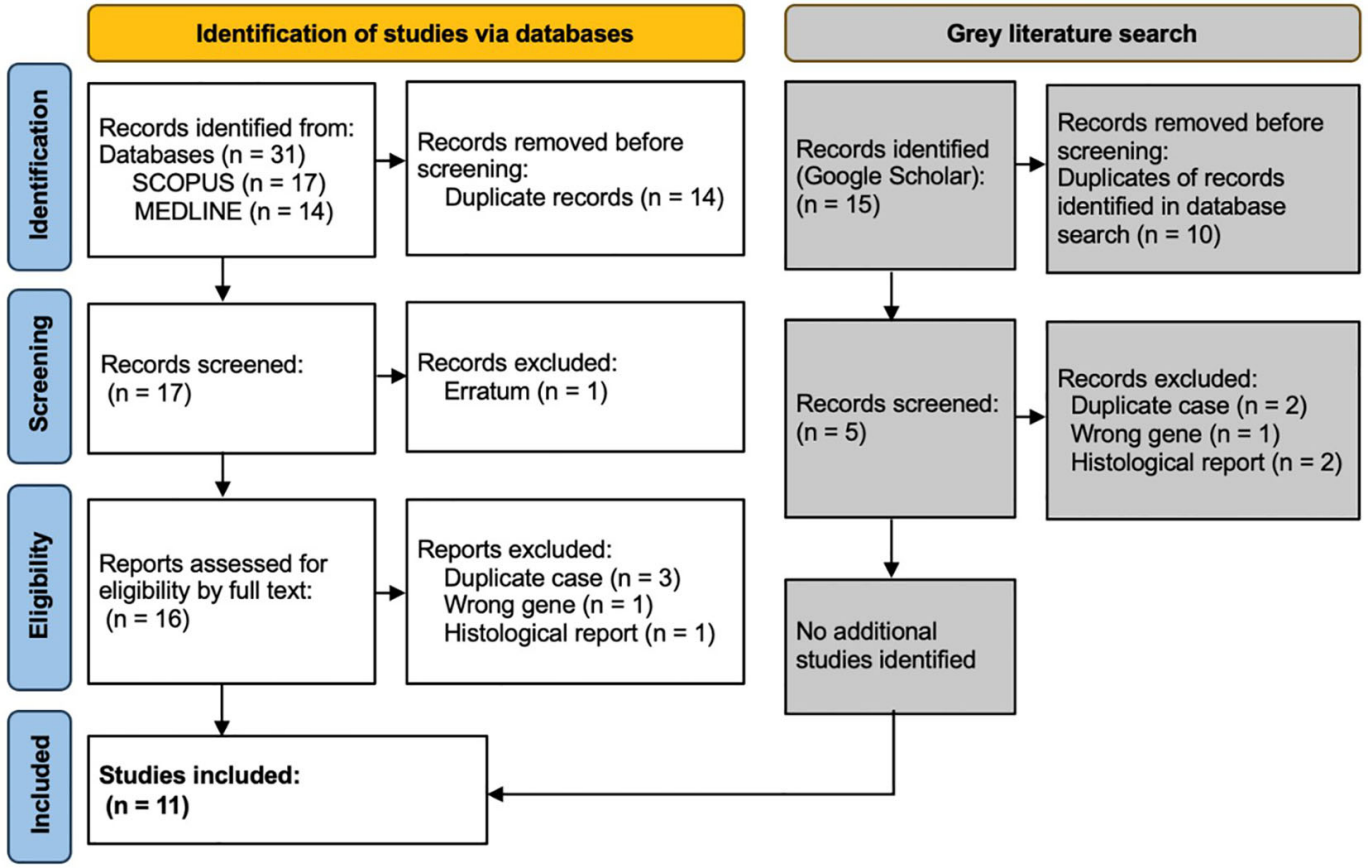
PRISMA diagram showing selection of studies for inclusion in scoping review. Template and process from Page et al. (28).

The search on Medline gave 14 results, of which 3 were excluded: one was a discussion about tumour histology, one was an *erratum* (author name spelling error), and one was regarding a different gene (*BARD1*). SCOPUS provided 13 results and 4 secondary documents. All 13 of these were included within the 14 results already found via Medline, and the same 3 papers were excluded. Within the 4 secondary documents, 1 was the 14th paper found via Medline, and 3 were the initial case reports of cases later discussed in more detail within the previously identified papers and thus represented duplications of cases. The grey literature search using Google Scholar found no additional case reports. This resulted in 11 papers included in the detailed review (**Figure 1**).

### Fanconi anaemia type S patient cohort

In total, we have identified 12 cases of biallelic *BRCA1* mutation, 10 in patients with an FA-S phenotype and 3 seemingly unaffected individuals (**Tables 1**, **2**). A human with biallelic *BRCA1* mutations was first reported in 1995, in a woman with breast cancer at age 32 and no other clinical features, described as homozygous for a high penetrance breast/ovarian cancer-associated mutation, but this report was subsequently found to be inaccurate (39). The case report suggested that homozygous *BRCA1* c.2800delAA mutation did not increase cancer risk beyond heterozygosity. However, this report was later found to be inaccurate due to experimental error – there was an amplification bias during genotyping which led to relative excessive amplification of the mutant allele (40). Subsequent re-sequencing confirmed that both mutant and wild-type alleles were present (29).

**Table 1:** FA-S Cohort: clinical characteristics and genetic information.

		Patient 1 (P1)	Patient 2 (P2)	Patient 3 (P3)	Patient 4 (P4)	Patient 5 (P5)	Patient 6 (P6)	Patient 7 (P7)	Patient 8 (P8)	Patient 9 (P9)	Patient 10 (P10)
***BRCA1* protein product**		p.D821Ifs*25/p.V1736A	p.S198Rfs*35/p.R1699W	p.C903* homozygote	p.W372* homozygote	p.W372* homozygote	p.L431* homozygote	p.L431* homozygote	p.C61G/p.R1699Q	p.S282Yfs*15/p.Y978*	p.W372*/ p.H1673del
**Karyotype & sex**		46 XX (female)	46 XX (female)	46 XX (female)	46 XX (female)	46 XX (female)	46 XY (male)	46 XX (female)	46 XX (female)	46 XY (male)	46 XX (female)
**Ethnicity**		–	Finnish	Brazilian	Arab	Arab	Turkish	Turkish	–	Romanian	–
**Consanguinity**		No	No	Yes – third cousins	Yes – degree not reported	Yes – degree not reported	Yes – degree not reported	Yes – degree not reported	No	No	–
**Relations to other patients**		None	None	None	Sibling of P5	Sibling of P4	Sibling of P7	Sibling of P6	None	None	None
**Age at last follow up**		29y (deceased)	25y (deceased)	6y (deceased)	5y (deceased)	6y	15y	7y	30y	2y	13m (deceased)
**Growth**	**At birth**	–	SGA	SGA	SGA	AGA	SGA	AGA	–	SGA	SGA
	**At assessment**	Short stature (−2.1 SD at adult height)	Short stature (−4.35 SD at adult height)	Short stature (−6.1 SD at 2.5y)	Short stature (−3.6 SD at 5y)	Failure to thrive	Short stature (Height <3%ile)	Short stature (Height <3%ile)	Short stature (−2.1 SD at adult height)	Short stature (−5.3 SD at 2y)	Small at 13m (weight −0.3 SD <3%ile, OFC –4.2 SD <3%ile)*
**Head & face**	**Microcephaly**	Yes	Yes	Yes	Yes	Yes	Yes	Yes	Yes	Yes	Yes
	**Jaw**	Macrognathia	Narrow palate, malocclusion	Micrognathia	Micrognathia	Micrognathia, triangular face	–	–	Triangular face	Micrognathia	Micrognathia, laryngotracheo-malacia
	**Eyes**	–	Upslanting palpebral fissures, blepharoph-imosis, epicanthus, strabismus, hypertelorism, microphthalmia	Upslanting palpebral fissures, long eye lashes	Upslanting palpebral fissures, microphthalmia	Upslanting palpebral fissures, epicanthus, microphthalmia	Microphthalmia	Microphthalmia	–	Upslanting palpebral fissures, epicanthus, strabismus	Left eye congenital cataract
	**Ears**	–	Conductive hearing loss	Ear abnormality	–	Cupped ears	–	–	–	Cupped ears.	Low set retrorotated ears
	**Other**	Low anterior hairline, prominent nasal bridge, small alae nasi.	Sparse hair, broad nasal bridge/tip	Bitemporal narrowing.	–	Short neck.	–	–	Low set ears, right-sided hearing loss.	Bitemporal narrowing.	Sparse hair, bitemporal narrowing, broad nasal bridge/tip, thickened alae nasi, anteverted nares, deep philtrum
**Skin**	**Hypopigmented**	–	Yes	No	Yes	Yes	Yes	Yes	No	Yes	No
	**Hyperpigmented^†^**	–	Yes	Yes	Yes	Yes	Yes	Yes	Yes	Yes	Yes*
**Intellectual disability**		Yes, undefined	Yes, mild	Yes, undefined	Yes, undefined	Yes, mild	Yes (IQ: 50-69)	Yes (IQ: 62)	–	Yes, mild	–
**Bone marrow failure**		No	No	No	No	No	No	No	No	No	No
**Other dysmorphisms & diagnoses**		–	Proximally inserted thumbs, 2nd digit camptodactyly, 2–3 toe syndactyly, duodenal stenosis, hip dislocation, enlarged left kidney	Clinodactyly, atrial septal defect	Small oedematous palms and soles, optic nerve hypoplasia, bilateral hip dysplasia	Small palms, right first rib hypoplasia and left 11 ribs	Hypoplastic thumb, right cryptorchidism, adrenal insufficiency, gliosis: periventricular and subcortical	Atrial septal defect, ventricular septal defect, growth hormone deficiency, gliosis, prominent cortical sulci	Left congenital hip dislocation, celiac disease	Clinodactyly, wide intermammillary distance, patent ductus arteriosus, micropenis, bilateral cryptorchidism	Wide intermammillary distance, bilateral transverse palmar crease, fifth finger brachydactyly, bilateral 2-3 toe syndactyly, hypoglycaemia
**Chromo-somal breakage**	**Spontaneous**	–	No	No	No	–	No	No	–	No	–
	**DEB-induced**	–	Yes	Yes	Yes	Yes	Yes	Yes	No	Yes	–
	**MMC-induced**	–	Yes	–	–	Yes	–	–	–	Yes	No^‡^
**Malignancy**		Stage IV papillary serous ovarian carcinoma (28y)	Ductal breast carcinoma (23y)	Diffuse astrocytoma (6y)	T cell acute lymphocytic leukaemia (5y)	None as of early 2020 follow-up	None as of early 2020 follow-up	Neuroblastoma (2y)	Invasive ductal breast carcinoma (30y)	None as of publication in 2020.	Malignant CNS tumour NEC, WHO grade 4 (13m)
**Sensitivity to chemotherapy**		Yes – hyper-sensitivity to carboplatin and paclitaxel (severe neutropenia, anaemia, and thrombopenia)	No – tolerated chemotherapy normally (docetaxel, fluorouracil-epirubicin-cyclophosph-amide)	– none given - died following decompressive neurosurgery	Unclear – poor response to methotrexate, prednisone and ½ dose vincristine (died after 2w; blast count 8%, hepatitis, enterococcal sepsis, varicella)	–	–	No – tolerated chemotherapy normally	Yes – hyper-sensitivity to (epirubicin/cyclophosph-amide (haemato-toxicity) and paclitaxel/carboplatin (thrombopenia, neutropenia, and anaemia)	–	– none given –palliated following neurological deterioration post-surgery.
**Family malignancy history**		Breast, ovarian, peritoneal and intestinal CA in 1st-4th degree relatives	Ovarian in 1st-2nd degree relatives, lung, endometrial, skin 2nd-3rd degree relatives	Breast cancer in 1st, 2nd and 4th degree relatives.	Intestinal and urological cancer, 2nd-3rd degree relatives	Intestinal and urological cancer, 2nd-3rd degree relatives	Uterine, oesophageal, and lung cancer, 2nd-3rd degree relatives	Uterine, oesophageal, and lung cancer, 2nd-3rd degree relatives	Breast and prostate cancer, 3rd degree relatives	Breast cancer in 3rd degree relatives	–
**Reference**		Domchek et al., 2014 (29)	Sawyer et al., 2015 (30)	Freire et al., 2018 (31)	Seo et al., 2018 (32)	Seo et al., 2018 (32)	Seo et al., 2018 (32)	Seo et al., 2018 (32)	Keupp et al., 2019 (33)	Chirita-Emandi et al., 2020 (34)	Borlin et al., 2022 (35)

AGA, appropriate for gestational age; SGA, small for gestational age; DEB, diepoxybutane; MMC, mitomycin C; %ile, percentile; SD, standard deviation; –, not reported; OFC, head circumference; y, year; m, months; CNS, central nervous system; NEC, not elsewhere classified.

*Data from personal correspondences.

^†^Including café-au-lait macules.

^‡^Mitomycin C-induced chromosomal breakage analysis in peripheral blood lymphocytes showed strongly reduced proliferation upon stimulation, but no evidence of increased chromosomal breakage (skin fibroblasts not available).

**Table 2:** Individuals reported to have biallelic BRCA1 mutations without any FA-like phenotype: clinical characteristics and genetic information.

	Patient N1 (PN1)	Patient N2 (PN2)	Patient N3 (PN3)
***BRCA1* protein product**	N/A, intronic mutation (spliceogenic variant) c.4096 + 3A>G homozygote	p.R1028C homozygote	p.N1355Yfs*10/p.D1778Gfs*27
**Karyotype & sex**	46 XX (female)	46 XX (female)	46 XX (female)
**Relations to other patients**	None	None	None
**Age at last follow up**	58y	62y	47y (deceased)
**Growth**	Normal height	–	Normal height
**Dysmorphisms/diagnoses**	Single café-au-lait macule	Large uterine polyp	Atrophic left kidney, pelvic stone, possible renal vein thrombus
**Intellectual disability**	No	–	–
**Malignancy**	None	Chronic lymphocytic leukaemia (48y), lobular carcinoma *in situ* (LIN2) (62y)	Stage IIIA serous ovarian adenocarcinoma (43y), invasive ductal breast carcinoma (44y)
**Sensitivity to chemotherapy**	–	No – tolerated chemotherapy normally	No – no hypersensitivity to carboplatin was noted, although response to chemotherapy was generally poor and progression to 4th line therapy was required
**Family malignancy history**	Breast and ovarian cancer in 2nd–4th degree relatives	Breast cancer in 1st-3rd degree relatives. Leukaemia in 1st degree relative	Breast, ovarian and cervical cancer in 1st degree relatives
**Bone marrow failure**	No	No	No
**Chromosomal breakage**	No (undefined test)	No (DEB induced)	–
**Reference**	Byrjalsen et al., 2017 (36)	Bondavalli et al., 2017 (37)	Kwong et al., 2021 (38)

DEB, diepoxybutane; –, not reported.

The first validated case of biallelic *BRCA1* mutations was identified in 2013 by Domchek et al., in a woman with ovarian carcinoma at age 28 (P1, **Table 1**) (29). P1 is a *BRCA1* compound heterozygote with a known deleterious mutation (p.D821Ifs*25) (41), in *trans* with a hypomorphic allele (p.V1736A). P1 also has a monoallelic *BRCA2* mutation (p.R324T), which is a variant of unknown significance (VUS). Prior to publication the p.V1736A allele was classified as a VUS, but once identified in P1 it was thoroughly investigated genetically and biochemically to ascertain whether this patient represented a genuine case of biallelic pathogenic *BRCA1* mutations. These investigations convincingly support the view that p.V1736A is a hypomorphic alteration affecting DNA repair (**Table 3**) (29). Interestingly, this patient was not originally suggested to have a form of FA, likely due to the absence of BMF. However, a number of clinical features differentiate P1 from a typical HBOC (BRCA1) phenotype. P1 experienced significant sensitivity to crosslinking chemotherapeutics (requiring discontinuation), and was reported to have short stature, microcephaly, facial dysmorphisms and intellectual disability. It is not known whether she had chromosome instability, as no breakage testing was undertaken prior to her death six months following cancer diagnosis.

**Table 3:** Detailed information regarding mutations found in individuals with biallelic *BRCA1* mutations.

*BRCA1* Protein	*BRCA1* Transcript	Patient & context	ClinVar ID	Clinvar analysis	Functional consequence of mutation	HBOC-associated?	Proposed viability mechanism
p.C61G	c.181T>G	P8 compound heterozygous with c.5096G>A in *trans*	17661	Pathogenic	Missense mutation resulting in loss of function, alters Zn^2+^-binding residue (42, 43), prevents interaction with BAP1 (BRCA1 associated protein-1) (44).	Yes (45, 46)	Combination of this deleterious allele with hypomorphic allele.
p.S198R fs*35	c.594_597del	P2 compound heterozygous with c.5095C>T in *trans*	209105	Likely pathogenic	Frameshift: predicted to result in premature termination within exon 11 (p.233X). However, this mutation occurs in an exon that is absent in the predominant, in-frame, naturally occurring isoform Δ9,10 and may be spliced out to produce a partially functional protein (47). RT-PCR analysis suggested increased nonsense-mediated mRNA decay. LOH analysis on genomic DNA from P2’s tumour showed no LOH at either BRCA1 allele – suggesting that both mutated alleles are dysfunctional (30).	Yes (46) but risk likely to be reduced compared with high penetrance mutations. High incidence of breast/ovarian cancer in relatives with mutation (30).	Combination of this hypomorphic allele with deleterious allele, partial rescue of this allele by Δ9,10 isoform.
p.S282Y fs*15	c.843_846del	P9 compound heterozygous with c.2933dupA in *trans*	17683	Pathogenic	Frameshift: predicted to result in premature termination within exon 11 (p.297X), causing loss of protein function through truncation or nonsense-mediated mRNA decay (48). This variant lies 3’ to alternative donor splice site in exon 11 at c.787 so would be deleted from the Δ11q minor transcript, which should be unaffected (32, 49).	Yes (46, 48, 50)	p.Y978* and p.S282Yfs*15 variants both lie 3’ to the splice site, hence two copies of the Δ11q minor transcript would likely be present (32, 49).
p.W372*	c.1115G>A	P4 and P5, homozygous, P10 compound heterozygous with c.5017_5019del in *trans*	54134	Pathogenic	Nonsense mutation predicted to cause truncation or total absence of the protein due to nonsense mediated decay. This variant lies 3’ to alternative donor splice site in exon 11 at c.787 so would be deleted from the Δ11q minor transcript, which should be unaffected (32, 49)	Yes (46)	In P4/5, homozygous truncating variant lies 3’ to the splice site, hence two copies of the Δ11q minor transcript would likely be present. In P10, one copy of the Δ11q minor transcript would likely be present, *in trans* with p.H1673del (32, 49).
p.L431*	c.1292T>G	P6 and P7, homozygous	54187	Pathogenic	Nonsense mutation predicted to cause truncation or total absence of the protein due to nonsense mediated decay. This variant lies 3’ to alternative donor splice site in exon 11 at c.787 so would be deleted from the Δ11q minor transcript, which should be unaffected (32, 49).	Yes (46)	Homozygous truncating variant lies 3’ to the splice site, hence the Δ11q minor transcript would likely be present (32, 49).
p.D821I fs*25	c.2457delC	P1 compound heterozygous with c.2457delC in *trans*. *BRCA2* p.R324T mutation (VUS)	37471	Pathogenic	Frameshift: predicted to result in premature termination (p.846X), causing loss of protein function through truncation or nonsense-mediated mRNA decay.	Yes (46)	Combination of this deleterious allele with hypomorphic allele, partial rescue of other allele by Δ11q isoform.
p.C903*	c.2709T>A	P3 homozygous	495221	Pathogenic	Nonsense mutation predicted to cause truncation or total absence of the protein due to nonsense mediated decay. This variant lies 3’ to alternative donor splice site in exon 11 at c.787 so would be deleted from the Δ11q minor transcript, which should be unaffected (32, 49).	Not reported	Homozygous truncating variant lies 3’ to c.787 splice site, hence the Δ11q minor transcript would likely be present (32, 49).
p.Y978*	c.2933dupA	P9 compound heterozygous with c.843_846del in *trans*	236270	Pathogenic	Nonsense mutation predicted to cause truncation or total absence of the protein due to nonsense mediated decay. This variant lies 3’ to alternative donor splice site in exon 11 at c.787 so would be deleted from the Δ11q minor transcript, which should be unaffected (32, 49).	Reported for a variant resulting in the same protein product (50)	Y978* and p.S282Yfs*15 variants both lie 3’ to the c.787 splice site, hence two copies of the Δ11q minor transcript would likely be present (32, 49).
p.R1028C	c.3082C>T	PN2 homozygous	37506	Likely benign	Missense mutation within exon 11, *in silico* models and clinical data predict this mutation to be benign (37).	Not reported to confer significant risk (51)	Homozygosity for a missense VUS.
p.N1355Y fs*10	c.4065_4068del	PN3 compound heterozygous with c.5406 + 7A>G in *trans*	17674	Pathogenic	Frameshift: predicted to result in premature termination within exon 11 (p.1365X), causing loss of protein function through truncation or nonsense-mediated mRNA decay.	Yes (46)	Combination of this deleterious allele with VUS, partial rescue of this allele by Δ11q isoform.
N/A	c.4096 + 3A>G	PN1 homozygous	37566	Uncertain significance	Spliceogenic variant located close to exon 11 donor splice site. *In silico* analysis predicts deleterious effect (destruction of the donor splice site) (36). *In vitro* RT-PCR demonstrates increase in Δ11 isoform and deletion of Δ3309nt 3′ of exon 11, with some normal residual transcript (52). Reported to display classical pathogenic characteristics whilst allowing homozygous viability (53).	Yes, reported as a Finnish founder pathogenic variant (54), pathogenicity is contested	Homozygosity for a missense VUS.
p.H1673del	c.5017_5019del (also known as 5136delCAC)	P10 compound heterozygous with c.1115G>A in *trans*	55355	Likely pathogenic	In-frame deletion of 3 nucleotides resulting in deletion of a histidine residue but preserves reading frame. *In silico* analysis shows that H1673 is in the BRCT domain predicted to interact with the BRCT domain of BARD1. Variant is absent in human variation databases. Loss of *BRCA1* WT-allele in 6 ovarian cancers and 2 breast cancers. 2,263,474:1 in favour of causality using Goldgar multifactorial likelihood method (55).	Yes, reported in ovarian & breast cancers in 14 Italian families, segregates with disease in related individuals (55).	Compound heterozygosity with p.W372* variant which lies 3’ to the c.787 splice site, hence one unaffected copy of the Δ11q minor transcript would likely be present (32, 49).
p.R1699W	c.5095C>T	P2 compound heterozygous with c.594_597del in *trans*	55396	Pathogenic	Missense mutation. Disrupts binding and transcriptional activity of BRCA1 (56).	Yes (46, 57)	Combination of this deleterious allele with hypomorphic allele, partial rescue of other allele by Δ9,10 isoform.
p.R1699Q	c.5096G>A	P8 compound heterozygous with c.181T>G in *trans*	37636	Pathogenic	Missense mutation. Decreases transactivation compared to WT in mammalian cells (56). Reduces embryonic stem cell survival, upregulates microRNA-155 (upregulated in many human cancers, WT BRCA1 downregulates microRNA-155) (58).	Yes, confers intermediate breast and ovarian cancer risk (59)	Combination of this hypomorphic allele with deleterious allele.
p.V1736A	c.5207T>C	P1 compound heterozygous with c.2457delC in *trans*. *BRCA2* p.R324T mutation (VUS)	37648	Pathogenic	Missense mutation resulting in loss of function, according to saturation genome editing assay, predicted to result in a hypomorphic allele (60). p.V1736A is within the first BRCT domain of BRCA1, a phospho-peptide recognition domain needed for binding to phosphorylated repair proteins. p.V1736A BRCT fragments demonstrated decreased localisation to DSB and reduced interaction with BRCA1-interacting protein RAP80 (29). LOH analysis of tumours with this variant demonstrated persistence of the mutated allele (in the ovarian tumour of P1 there was no LOH, suggesting that no selective pressure to lose either allele). The p.V1736A positive side of the family (maternal) displayed a pedigree consistent with HBOC (BRCA1), including individuals with: ovarian cancer age <60, ovarian and bilateral breast cancer, peritoneal cancer (all p.V1736A positive and p.846X negative)^†^. Additionally, mouse studies have demonstrated that homozygous mutations in the BRCT regions yield viable animals with increased cancer risk (61).	Yes, including multiple family members, but risk is likely to be reduced compared with high penetrance mutations as allele is hypomorphic (29). 11 additional families with heterozygous p.V1736A mutations have been identified, combined odds ratio for pathogenicity found to be 234:1 (29)	Combination of this hypomorphic allele with deleterious allele, partial rescue of this allele by Δ11q isoform.
p.D1778G fs*27	c.5406 + 7A>G	PN3 compound heterozygous with c.4065_4068delTCAA in *trans*.	55566 (cDNA variant), 993167 (protein variant)	Likely benign (cDNA variant). Pathogenic (74bp del resulting in protein variant)	Splice site variant in intron 22, frameshift mutation, allele predicted to be functional by saturation genome editing (60). PN3 found to have deletion of 74 nucleotides (r.5333_5406del74) resulting in the pathogenic protein product, which causes frameshift termination and deletion of exon 22 (38).	Yes (38), including sister of PN3.	Combination of this VUS with deleterious allele, partial rescue of other allele by Δ11q isoform.

HBOC, hereditary breast and ovarian cancer; LOH, loss of heterozygosity; VUS, variant of unknown significance; RT-PCR, Reverse transcription polymerase chain reaction; UTR, untranslated region; DSB, double-strand breaks; WT, wild-type; del, deletion; fs, frameshift. ClinVar: https://www.ncbi.nlm.nih.gov/clinvar/.

^†^The BRCA2 VUS p.R324T appears on this side of the family but does not appear to segregate with disease – the individual with ovarian and bilateral breast cancer is BRCA2 p.R324T negative, supporting the view that it may not be of clinical significance (29).

Sawyer et al. defined the FA-S subtype with the identification of P2 in 2015 (30). She presented with multiple congenital abnormalities including short stature, microcephaly, facial dysmorphisms, hypo- and hyper-pigmented skin lesions, proximally inserted thumbs (radial ray anomaly) and intellectual disability, as well as ductal breast carcinoma aged 23 (P2, **Table 1**). She had originally been suggested to have Dubowitz syndrome and was genotyped as part of an effort to identify Dubowitz-associated genes, during which she was found to have compound heterozygous *BRCA1* mutations. A p.R1699W missense mutation, previously identified as pathogenic in HBOC (BRCA1) families (56), as well as a p.S198Rfs*35 mutation. DEB and MMC testing showed elevated chromosome breakage within diagnostic parameters for FA. Despite the chromosomal breakage results, she tolerated chemotherapy without signs of haematotoxicity. P2 has recently been discussed in a paper considering alternative genomic diagnoses for patients clinically diagnosed with Dubowitz syndrome but re-diagnosed with other disorders following genomic testing (62).

Freire et al. presented the first patient with homozygous pathogenic *BRCA1* mutations – a girl (P3, **Table 1**) aged 2.5 years with similar dysmorphic phenotypic presentation to P1/P2 (31). P3 was found to have a homozygous nonsense *BRCA1* mutation (p.C903*) resulting in premature termination within exon 11 and loss of key protein functional domains. Although this mutation has not been identified as an HBOC-associated variant, loss-of-function truncating mutations distal of this site have been reported as pathogenic, strongly suggesting pathogenicity (63). Cytogenic testing showed increased DEB-induced chromosomal breakage. Upon investigation, P3’s mother (p.C903* heterozygote) was found to have undifferentiated metastatic adenocarcinoma (31). P3 was not reported to have any malignancy in the original paper. Unfortunately, following publication, P3 developed a diffuse astrocytoma and died shortly following decompressive neurosurgery (64).

Following this, four patients (two pairs of siblings) with homozygous *BRCA1* nonsense mutations were identified by Seo et al. – one pair with biallelic p.W372* mutations (P4 and P5, **Table 1**) and the other pair with biallelic p.L431* mutations (P6 and P7, **Table 1**) (32). These patients all presented with microcephaly, microphthalmia, abnormally pigmented skin lesions, intellectual disability and growth abnormalities (short stature or failure to thrive), as well as elevated chromosomal sensitivity to DEB and/or MMC. Interestingly, siblings P6/P7 presented with endocrine and neuroanatomical anomalies, which are not observed in any other FA-S patients to date and are not typical of FA. This may be a result of their particular *BRCA1* mutation, or alternately caused by a separate undiagnosed condition. Endocrine abnormalities are common in FA, including growth hormone deficiency and hypothyroidism, but CNS anomalies present in only around 8% of FA cases (1). P4 and P7 developed childhood cancers, T-cell acute lymphocytic leukaemia (ALL) and neuroblastoma respectively. P7 responded normally to chemotherapy. However, P4 responded poorly to chemotherapy, even with a reduced-dosage regime. Although a haematotoxic response was not reported, P4 died soon after initiation of chemotherapy as a result of complex infections (**Table 1**). This might be suggestive of severe haematotoxicity and could represent a second patient in the cohort with sensitivity to crosslinking chemotherapeutics. As of an early 2020 follow-up, the status of patients P5-7 remains the same as at the time of publication (personal correspondence, 2021).

Three further patients have since been identified (P8, P9 and P10, **Table 1**). P8 was a compound heterozygote with two pathogenic *BRCA1* mutations (33) – one high penetrance (p.C61G) (45) and one conferring intermediate risk (p. R1699Q) (59). P8 has congenital abnormalities consistent with previously identified FA-S patients, an early breast cancer (age 30), and had a haematotoxic response to crosslinking chemotherapeutics. Surprisingly, P8 did not exhibit DEB-induced chromosomal instability, and it is therefore disputable whether she should be considered a canonical FA-S patient. However, she is included here due to the burden of FA-S-associated clinical signs and lack of MMC testing. P8 does appear to have the mildest phenotype of FA-S patients (and is the oldest surviving FA-S patient to-date), but should certainly be distinguished from classical HBOC (BRCA1) patients on account of the congenital abnormalities, sensitivity to crosslinking chemotherapeutics, and presence of two pathogenic mutations in *trans*. P9 is the second male patient to be identified with FA-S (34), and is a compound heterozygote with two pathogenic truncating *BRCA1* mutations in *trans*: p.S282Yfs*15 and p.Y978*. He presented with a phenotype similar to previous FA-S patients (growth restriction, multiple dysmorphic facial features and skin pigmentation) and exhibits chromosomal sensitivity to DEB and MMC, but no malignancy has been reported.

The most recently identified FA-S patient, P10, was a female compound heterozygote with a pathogenic mutation p.W372*, in *trans* with a p.H1673del mutation (35); the p.W372* mutation being the same as P4/5, and also reported as HBOC-associated (46). Genetic analysis strongly supports the view that p.H1673del is a pathogenic mutation, with association with an ovarian-predominant HBOC presentation in heterozygotes, loss of heterozygosity (LOH) in breast/ovarian cancers, and segregation with affected individuals in families (55). *In silico* modelling predicted an effect on BARD1 binding to the BRCT region, and multifactorial likelihood calculation gave a very high ratio in favour of causality (**Table 3**). P10 presented with multiple dysmorphic features including laryngotracheomalacia, abnormally pigmented skin lesions, distal skeletal abnormalities, and growth abnormalities. She developed a malignant CNS tumour (not elsewhere classified, WHO grade 4) at 13 months, and died 6 weeks later following a palliative approach to treatment because of rapid tumour progression of the tumour. Although there was no evidence of increased MMC-induced chromosome breakage in lymphocytes, stimulation resulted in strongly reduced proliferation. Despite compound heterozygosity, P10 had a severe phenotype (in terms of both dysmorphisms and an early childhood cancer) aligning more closely with the homozygous patients described so far.

### Biallelic *BRCA1* mutations without FA-like disorder

At least three individuals (PN1-N3, **Table 2**) have been reported with biallelic *BRCA1* mutations without an FA phenotype. PN1 has a homozygous spliceogenic variant predicted to be highly deleterious, however PN1 has no clinical features consistent with FA or HBOC (BRCA1), suggesting that this variant is likely benign (36). PN2 has a homozygous missense mutation (p.R1028C) predicted to be ‘likely benign’ and has no congenital abnormalities – although chronic lymphocytic leukaemia and breast cancer were reported, suggesting a possible HBOC (BRCA1) phenotype (37). PN3 is a compound heterozygote with breast/ovarian cancers before age 50, but no congenital anomalies – a phenotype more consistent with HBOC (BRCA1) than FA (38). One allele is a known pathogenic mutation (p.N1355Yfs*10), co-occurring with a splice site variant with complex interpretation (p.D1778Gfs*27, **Table 3**). The sister of PN3 (p.D1778Gfs*27 heterozygote) had breast/ovarian cancers at around 10 years older than the age PN3 developed cancer, possibly suggesting a worsened HBOC (BRCA1) phenotype due to compound heterozygosity.

## Discussion

The emergence of a cohort of patients with pathogenic biallelic *BRCA1* mutations and an FA-like phenotype offers insights into the developmental role of *BRCA1*, as well as broadening the definition of the FA phenotype. There are a number of outstanding questions regarding the underlying biochemistry and pathophysiology of FA-S, as well as a lack of guidance for optimal clinical management. These are likely to be addressed as additional FA-S patients are identified – FA-S may be underreported currently, as FA panels do not typically screen for *BRCA1*.

It is therefore important that patients with very early breast/ovarian cancer and congenital abnormalities, as well as patients with an FA-like phenotype (particularly in the absence of BMF), are screened for biallelic *BRCA1* mutations. Prior to genotyping, many of the FA-S patients described here were investigated for differential diagnoses to explain observed chromosome instability. These include Dubowitz syndrome, non-specific FA, Nijmegen breakage syndrome (NBS), Bloom syndrome and ataxia telangiectasia (AT) (34, 65). NBS, Bloom syndrome and AT are chromosome instability syndromes resulting in cancer predisposition and congenital anomalies, however immunodeficiency is usually present in these cases (unlike in FA) (1, 65). Given the phenotypic variability of FA-S, patients with atypical presentation of these conditions who have not been genetically diagnosed should be considered for *BRCA1* testing. Furthermore, the emergence of FA-S as a clinical syndrome may have implications for genetic counselling in families with HBOC (BRCA1). Currently, there is no clear boundary between FA-S and BRCA1-associated hereditary breast and ovarian cancer (HBOC), as exemplified by PN2-3 and P8. These patients are harder to categorise as they have breast and/or ovarian cancer but no evidence of increased chromosomal breakage and minimal dysmorphisms. Presently, these cases are segregated predominantly by their underlying genetics as well as the phenotypic severity (age of cancer, sensitivity to chemotherapeutics and congenital dysmorphisms), however, future study of the functional consequences of these mutations may lead to recategorization. P8 and PN3 are the most borderline cases in terms of both genotype and phenotype and both cases underline that these conditions lie on a continuous spectrum of clinical disease.

**Figure 2:**
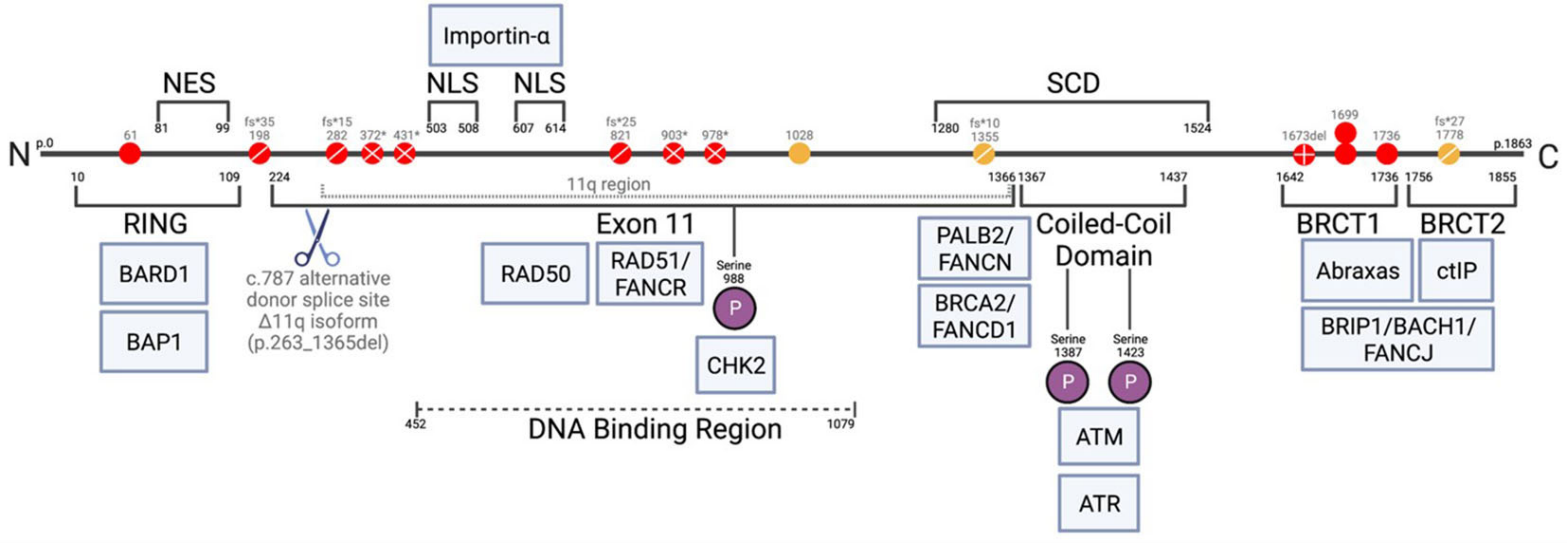
Schematic of full-length (1863 amino acid) BRCA1 protein, with c.787 alternative donor splice site and 11q region (66, 67). Alternative splicing at c.787 resulting in p.263_1365del from protein, Δ11q isoform. Mutations found in FA-S patients shown as red circles, mutations found in patients PN1-3 shown as yellow circles: ◯; missense, ⊘; frameshifts, ⊗; nonsense, ⊕; deletion. Associated proteins shown in blue at approximate points of interaction with BRCA1. RING, zinc finger; NES, nuclear export signal; NLS, nuclear localization sequence; SCD, serine cluster domain; BRCT, BRCA1 C-terminal; P, phosphate.

One controversy regarding FA-S was the expected lethality of biallelic *BRCA1* mutations. The mutations found in this cohort are described in detail in **Table 3**, including evidence for functional consequences of these mutations, associations with disease in the heterozygous setting (HBOC), and proposed viability mechanisms in these patients. Within the main cohort P1-10, nine out of ten have at least one mutation that could be partially rescued by alternative splicing. One of these (P2) may be rescued by the presence of the naturally occurring Δ9,10 isoform. The remaining eight (P1, P3-7, P9-10) each have at least one mutation that may be rescued by the Δ11q isoform, as proposed by Seo et al., in reference to the homozygous mutations seen in P3-P7 (31, 32). All of the mutations seen in P3-7 lie within, or 3’ of, an alternatively-spliced region, hence allowing unaffected translation of a naturally occurring minor transcript – Δ11q (**Figure 2**). Δ11q is approximately 40% the length of full-length BRCA1 and consists of normal 5’ and 3’ untranslated regions with truncated exon 11 (c.788_4096del), retaining the reading frame and yielding a shortened, but partially functional, protein isoform (p.263_1365del) (32, 49). The Δ11q isoform has also been shown to underlie a mechanism of resistance to PARP inhibitors and cisplatin in the management of *BRCA1* mutated cancers (49). Δ11q isoforms were significantly enriched in fibroblasts from P5 relative to full-length transcripts (compared with control fibroblasts), as a result of nonsense-mediated decay (NMD) of the full-length transcript (32). PN1 and PN2 also have homozygous *BRCA1* mutations within the truncated region of exon 11, that could therefore be partially rescued by alternative splicing. However, these may simply be non-pathogenic mutations - the mutation found in PN1 is a spliceogenic VUS with contested pathogenicity, and the mutation in PN2 is not reported to confer significant risk in a heterozygous setting and predicted to be benign. Hence, PN1 and PN2 are not included in the main cohort here – although PN2 could be considered to have an HBOC-like phenotype.

With regards to compound heterozygous *BRCA1* mutations in FA-S patients, most appear to be compatible to life because a hypomorphic missense allele co-occurs with a highly deleterious allele (P1, P2 and P8) hence enough function is retained to enable embryonic survival (29, 30). Previously, it has been considered that the co-occurrence of a VUS in *trans* with a known pathogenic *BRCA1* mutation suggests that the VUS must be benign (68), however the existence of these FA-S patients with viable biallelic mutations challenges this view and has significant implications for how *BRCA1* VUS are interpreted. The hypomorphic variants found in *trans* with highly deleterious mutations in FA-S compound heterozygotes likely confer reduced HBOC risk in a heterozygous setting, as has been suggested for p.V1736A and p.S198Rfs*35 (30) (**Table 3**). However, alternative splicing may also allow for viability in these cases, as the hypomorphic alleles found in both P1 and P2 can be partially rescued by alternative splicing (Δ11q and Δ9,10 respectively). PN3 is a compound heterozygote with a known deleterious allele in *trans* with a VUS, whereby the deleterious allele is within the truncated region of exon 11 allowing for a normal Δ11q isoform. PN3 can be confidently considered to have an HBOC-like phenotype, however is not included in the FA-S cohort due to the second mutation being a VUS with unclear pathogenicity, and an overall lower burden of disease compared with P1-10.

P8 is the only case where neither mutation is rescuable by alternative splicing. Despite this, the phenotype of P8 is unexpectedly mild, especially given the known deleterious mutations present in both the RING domain and BRCT domain. It is possible that an alternative rescue mechanism such as interallelic complementation may provide the explanation for this reduced severity phenotype. P8 is tentatively included in the main cohort due to the presence of in *trans* pathogenic mutations independently associated with HBOC when present in heterozygotes, as well as mild congenital dysmorphisms, sensitivity to chemotherapeutics and having a younger cancer and overall higher severity phenotype compared with PN2-3.

In the case of P9, both variants result in premature termination, however both are 3’ to the alternative donor splice site in exon 11, likely preserving the Δ11q minor transcript. Follow-up with P9 will ascertain whether his phenotype tends towards more severe (as in homozygotes) or less severe (as in most other compound heterozygotes). P10 is an interesting case, as one variant is a truncating mutation 3’ to the splice site (allowing unaffected translation of the Δ11q minor transcript as described above). However, the other variant is downstream of exon 11 (exon 15) so would be included in the Δ11q isoform, likely resulting in NMD of the protein product of this allele. This might explain the severe phenotype seen in P10, which is similar to the most severe homozygotes, as only one allele can produce the partial rescue provided by the Δ11q minor transcript. This is in contrast to homozygotes, where both alleles are able to yield an unaffected Δ11q isoform. Hence, a Δ11q gene dosage effect might underlie the arguably more severe phenotypic presentation in P10 compared to the patients with homozygous mutations.

An interesting observation from the FA-S cases is the presence of breast and ovarian cancers within the cohort. Typically, females with FA do not get breast/ovarian cancers – malignancies are usually haematological (particularly acute myeloid leukaemia and myelodysplastic syndrome) (13), with a smaller proportion of solid (often embryonal) tumours (69), and a high incidence of squamous cell carcinomas in FA patients who reach their third-fourth decade of life (65). There are a number of suggested explanations; females with typical FA tend to be hypogonadal with low serum estrogen and reduced breast/ovarian tissue mass which may be protective against breast/ovarian tumours (70, 71). Additionally, many patients with FA die at a young age, before an age at which breast/ovarian tumours are likely to develop (45% of individuals with FA die from haematological complications before the age of 20) (71, 72). FA-S females may not be hypogonadal, as this was not reported in any of the seven female cases. However, hypogonadism in females is often not reported until teenage years with late puberty, or in milder cases not until presentation of fertility issues.

However, it should be noted that only the female compound heterozygous FA-S patients have presented with breast/ovarian tumours. The female homozygous FA-S patients are all younger, but 3/4 have presented with childhood cancers that align with a more typical FA presentation (astrocytoma, acute lymphoblastic leukaemia and neuroblastoma). P8, the patient with the mildest FA phenotype, is the only FA-S patient reported to have given birth (twin boys). It is unknown whether any fertility treatment was required. All female homozygous FA-S patients are still too young to have been expected to start puberty, and two have died as a result of malignancies. It would be valuable to ascertain whether the surviving female homozygotes (P5 and P7) present with symptoms of hypogonadism. Further to this, given that the homozygous FA-S patients seem to present with a more severe phenotype and ‘FA-like’ cancers, it would be of interest to follow-up with P5 and P7 to investigate whether they are later predisposed to breast/ovarian cancers [as in HBOC (BRCA1)] or not (as in other FA types).

Male hypogonadism does appear to be a feature of FA-S: both males in the cohort presented with cryptorchidism, P9 also with micropenis and low anti-Müllerian hormone. Only 2/10 FA-S patients identified thus far are male (and 0/3 of the non-FA-patients PN1-PN3). With only 10 patients in total this is likely to be coincidental, however possible explanations should be considered in case this observation continues as further FA-S cases are identified. One possibility is that FA-S males with compound heterozygous mutations are at a lower cancer risk than females (as compound heterozygous females seem to develop breast/ovarian cancer) so therefore are not identified. It can be expected that they would also have congenital abnormalities, but in the absence of BMF or malignancy this could easily be misdiagnosed. It is of note that the only male with a homozygous *BRCA1* mutation (P6) remains cancer-free despite reaching his late-teens – the oldest homozygous FA-S patient to do so. Another plausible explanation for the relative lack of male FA-S patients could relate to relative viability of male versus female embryos with *BRCA1* mutations. Sex ratio distortions (reduced proportion of males) have been reported in the offspring of *BRCA1* heterozygous females (73, 74), although the presence/extent of this distortion is disputed (75).

Another striking difference between FA-S patients and classical FA is the absence of BMF, as well as the relative rarity of radial anomalies (only seen in P2 and P6). Although BMF has been considered a hallmark of Fanconi Anaemia, FA-S is not the only type which does not exhibit it - case reports often refer to patients with these presentations as having an ‘FA-like disorder’. Another recently identified rare subtype, FA-O, appears to present similarly to FA-S – including congenital dysmorphisms, sensitivity to crosslinking agents, and without BMF, although only one consanguineous family and one additional individual have been identified (76, 77). In additional, with biallelic FANCM mutations present with an HBOC-phenotype, alongside sensitivity to chemotherapy and possible chromosomal sensitivity but without BMF or congenital abnormalities leading to significant dispute regarding the status of FANCM as a canonical FA protein, as patients (78). Even among more common types of FA, the presentation of BMF is variable – with estimated cumulative incidence of BMF at 10 years ranging from 12.6%-84% depending on predicted risk group (79).

**Figure 3:**
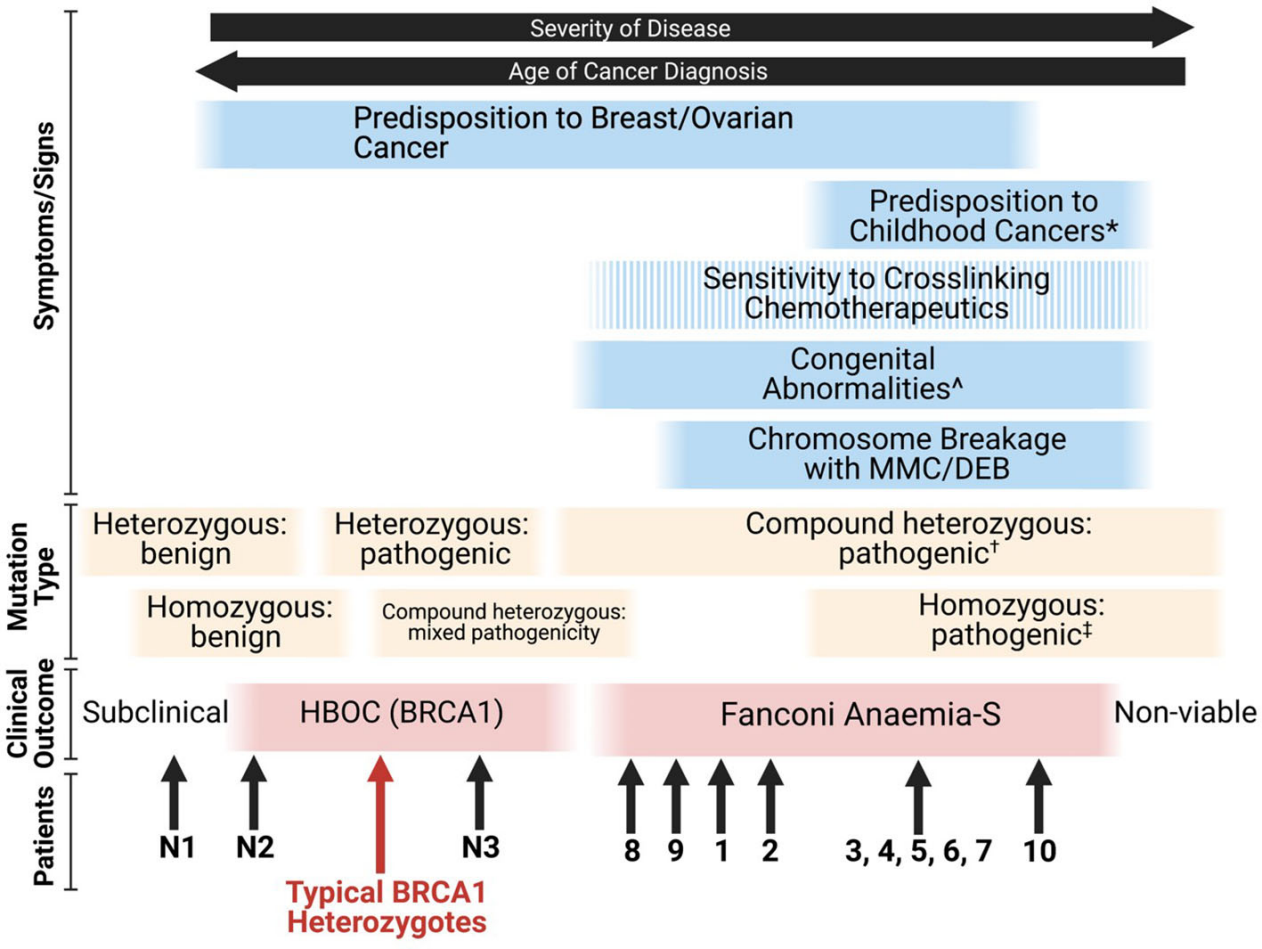
Proposed phenotypic spectrum of *BRCA1* mutations (67). ‘Sensitivity to cross-linking chemotherapeutics’ is a non-ubiquitous presentation among FA-S patients. *; ALL, neuroblastoma and astrocytoma are the three most frequent cancers in children (80). ^; short stature, microcephaly, head/facial dysmorphisms, pigmented skin lesions and intellectual disability. †; two highly deleterious alleles likely result in non-viability, for viability it appears that at least one partially functioning allele is required (or functioning splice variants of at least one allele). ‡; many homozygous pathogenic variants are likely to be non-viable, splice variants may account for viability.

Given that many combinations of deleterious *BRCA1* mutations are likely to be embryonically lethal (26), the FA-S phenotype appears to be an intermediate between classical HBOC (BRCA1) and non-viability. The phenotypic spectrum of *BRCA1* mutations stretches from subclinical benign monoallelic mutations through to pathogenic homozygous mutations resulting in nonviability of embryos, with some patients blurring the lines between FA-S and HBOC (BRCA1) (**Figure 3**). From the patient data available, it appears that homozygous mutations tend to result in a more severe FA-like phenotype, whereas compound heterozygosity results in a severe HBOC-like cancer phenotype along with congenital abnormalities. The Δ11q isoform appears to be a key mechanism for survival of biallelic *BRCA1* mutations particularly in a homozygous setting, and it is plausible that other splice variants may be subsequently found to provide alternative survival mechanisms.

## Author contributions

TH: Data curation, Investigation, Methodology, Writing – original draft. AR: Data curation, Formal Analysis, Writing – review and editing.
